# Exploring the nanofiltration mass transfer characteristic and concentrate process of procyanidins from grape juice

**DOI:** 10.1002/fsn3.1045

**Published:** 2019-04-26

**Authors:** Cunyu Li, Yun Ma, Hongyang Li, Guoping Peng

**Affiliations:** ^1^ College of Pharmacy Nanjing University of Chinese Medicine Nanjing China; ^2^ Jiangsu Collaborative Innovation Center of Chinese Medicinal Resources Industrialization Nanjing China; ^3^ The Forth Hospital of Taizhou City Taizhou China

**Keywords:** concentrate process, grape juice, mass transfer, nanofiltration, procyanidins

## Abstract

In order to separate procyanidins from grape juice at room temperature, a separation prediction model was established based on nanofiltration. The mass transfer coefficient was positively correlated with the initial concentration. Nanofiltration performance of procyanidins was affected by filtration conditions, membrane properties, and molecular states. The correlation between mass transfer coefficient and initial concentration was established based on the linear equations of the rejection and mass transfer coefficient. The rejection of procyanidins predicted with the mass transfer model was in accordance with the experimental value, and the antioxidant activity was preserved effectively. The mathematical model could predict the rejection of procyanidins. The nanofiltration technology for procyanidin separation from grape juice was characterized by fast separation, low energy consumption, and zero oxidization loss. The nanofiltration technology could greatly improve the utilization efficiency of food products and decrease the energy consumption.

**Practical application**

Thermal breakage of phenolic ingredients was a common problem to which attention should be paid in the application of food and chemistry industry. It has been evidenced that NF separation was an effective technique for the concentrate of procyanidins from the grape juice. Given today’s green separation demand over the world, it is important for the researchers to understand this method and its benefits for food and chemistry industry.

## 1 INTRODUCTION

Grape juice is a healthy drink. Phenolic compounds are the main active ingredients in grape juice (Revilla & Ryan, 2000). Phenolic compounds are important substances with complex structures, such as procyanidins, catechuic acid, and vitamins (Kyraleou et al.., 2015; Lecce et al., 2014; Montealegre, Peces, Vozmediano, Gascueña, & Romero, 2006). The concentration efficiency of phenolic compounds from plant materials depends on several parameters, such as temperature, time, and pressure. When the thermal concentration process is applied to obtain active constituents from grape juice, oxidation reactions, polymerization reactions, and isomerization reactions happen simultaneously (Dumpler & Kulozik, 2016; Imbierowicz, Troszkiewicz, & Piotrowska, 2015; Khanal, Howard, & Prior, 2010), thus leading to severe waste.

Nanofiltration (NF) is a pressure‐based membrane separation technology which uses nanoporous membranes and has a cutoff of 100–2,000 Da. NF has been developed as a potential technology for retaining food and medicine ingredients from water extraction without heat effect and shows high rejection, high water permeability, and moderate pressure requirements (Guu & Zall, 1992; Li, Qi, Luo, Khan, & Wan, 2015; Maher, Sadeghi, & Moheb, 2014; Lim, Scholes, Dumée, & Kentish, 2014). NF application in fruit juice processing is still in its infancy, and there are many theoretical and technical problems to be solved. Mathematical models have been optimized to predict NF membrane performance for compound separation. The behavior and rejection mechanisms of fruit juice phenolic compounds were analyzed based on the quantitative structure–activity relationship model (Hidalgo et al., 2013; Banerjee & De, 2011). However, the effect of molecular state on the performance mass transfer process was not discussed. In order to clarify the relationship between membrane transport mechanisms and molecular state, the mass transfer mathematic model was fitted and verified based on the solution–diffusion effect and Donnan steric partitioning pore model (Pérez, Escudero, Arcos‐Martínez, & Benito, 2016; Wang et al., 2012). Procyanidins were selected as the indication of phenolic compounds in grape juices to evaluate the performance of a NF membrane under different concentrations and pH. The prediction model of nanofiltration separation provides the prediction basis for nanofiltration separation, especially for functional food with phenolic compounds.

## 2 MATERIALS AND METHODS

### 2.1 Preparation of grape juice

Fresh grapes were obtained from a local market, which were from Pakwachow Island in Nanjing. The grapes were washed with purified water, and fruit branches were cut off. The grapes were processed in a commercial juicer to yield the natural juice. The natural juice was kept at 4–7°C to prevent damage or degradation.

### 2.2 Microfiltration pretreatment

In order to improve the clarity, grape juice was pretreated by microfiltration to remove suspended solids. In the microfiltration, a polyethersulfone (PES) membrane with a pore diameter of 0.2 μm and a filtration area of 0.3 m^2^, and max cross‐flow operation pressure of 0.80 MPa (Synder Filtration, USA) was used. The model of PES membrane was spiral, and the mode code was V0.2‐2B‐1812. A variable‐speed gear pump (Model JDB‐12A, Tuozhu Corporation, China) was used to circulate the feed solution, which can provide the constant flux at different feed pressure. The microfiltration conditions were set as 20 ± 2°C and 0.3 MPa in the experiment.

### 2.3 Procyanidin content

The content of procyanidins was determined with an Agilent 1,100 HPLC system equipped with a reverse‐phase column (Agilent C_18_, 4.6 mm Ø × 250 mm) at 30°C and a UV‐visible detector (*λ* = 280 nm). An isocratic mobile phase of 0.4% aqueous phosphoric acid: acetonitrile (15:85, ml/ml) mixture was used under a flow rate of 0.8 ml/min. The injection volume was 10 μl. For the quantitative analysis, a standard calibration curve was obtained by plotting the peak area against different concentrations (5, 10, 50, 150, 300 µg/ml) of procyanidin standard compound. The curves showed a good linearity and followed Beer’s Law (*r* ^2^ = 0.9987). Similarly, the final concentration of compounds in the samples in three consecutive injections was determined as the average content.

### 2.4 The Nanofiltration system and operations

A laboratory bench scale cross‐flow NF apparatus was used in all experiments. The apparatus consisted of a NF membrane, one variable‐speed gear pump (Model JDB‐12A, Tuozhu Corporation, China) for pressure and recirculation, a digital pressure gauge (Mettler Toledo, Germany) for the measurement of operating pressure, and tubings. The model of NF membrane was spiral, and the mode code was NFG‐2B‐1812. NF was carried out by using polyamide membrane with a molecular weight cutoff of 800 Da, a filtration area of 0.30 m^2^, max cross‐flow operation pressure of 3.0 MPa, PEG800 minimum rejection of 95.0%, and permeation flux of 76.5–93.5 L/(m^2^ h) (Synder Filtration, USA).

In order to ensure that the separation performance of the membranes was not changed during filtration experiments, first, remaining water was pumped from the NF apparatus. Second, microfiltered grape juice was used in NF system. Testing pressures were 0.2, 0.4, 0.6, 0.8, 1.0, and 1.2 MPa, and the permeate flux (*J*) was regulated by the variable‐speed gear pump. The pipeline of feed solution, filtrate, and rejected solution was placed in the same tube. Before sampling analysis, membrane module was pressurized at the test pressure for minimum 2 hr to reach the steady‐state conditions. When the adsorption–desorption equilibrium between solutes and membrane was reached, the concentrations of the feed and permeate were analyzed with high‐performance liquid chromatography (Agilent 1100, USA). And the rejection was calculated according to Equation (1) (Qiu & Yang, 2010).

(1) $$\text{Rejection}\;\left(\text{\textbackslash{}\%}\;\right)=\left(1-\frac{C_{\text{p}}}{C_{\text{f}}}\right)\times 100\text{\textbackslash{}\%}$$

where *C*_f_ and *C*_p_ are the solute concentrations in feed and permeate solution. Each measurement was performed in triplicate.

### 2.5 Nanofiltration separation prediction model

The solution–diffusion model of the NF assumes that the solute contacts the solvent and is dissolved on the membrane surface (Murthy & Gupta, 1997; Geraldes, Semião, & Pinho, 2001). Then, the solute passes through NF membrane pore under chemical potential differences. The model can be expressed as:

(2) $$J_{\text{V}}=L_{\text{p}}\left(p-\Delta \pi \right)$$

(3) $${\text{N}}_{\text{A}}=\frac{\mathrm{DK}}{\delta }\cdot \left(C_{\text{m}}-C_{\text{P}}\right)$$

where *J*_V_ is permeate flux, L/(m^2^ h); *L*_p_ is the pure water permeability, L/(m^2^ h Pa); *p* is operating pressure, Pa; Δπ is the osmotic pressure difference across the membrane, Pa; *K* is partition coefficient; *δ* is membrane thickness, cm; *DK/δ* is the mass transfer performance of a membrane, cm/s; N_A_ is the volume flux of solute, mol/(cm^2^ s); *C*_m _is solute concentrations in NF membrane surface, mol/L.

The rejection of solutes can be divided into apparent rejection *R_o_* and real rejection *R_r_*, which can be, respectively, expressed as:

(4) $$R_{\text{o}}=\frac{C_{\text{o}}-C_{\text{p}}}{C_{\text{o}}}$$

(5) $$R_{\text{r}}=\frac{C_{\text{m}}-C_{\text{p}}}{C_{\text{m}}}$$

*C*_o_ is the original solute concentration. Based on the solution–diffusion model and Equation (2) ‐ Equation (5), the relationship between *R*_o_ and mass transfer coefficient *k* can be expressed as:

(6) $$\ln\left[\left(1-R_{\text{o}}\right)\;\cdot J_{\text{V}}/R_{\text{o}}\right]=\ln\left[DK/\delta \right]+\frac{J_{\text{v}}}{k}$$

According to Equation (6), the relations between ln[(1‐*R*_o_)·*J*_v_/*R*_o_] and *J*_v_ are depicted by means of the linear fit. 1/*k* is the slope, and ln[*DK*/*δ*] is the intercept. The NF separation prediction model (25°C) was established with a series of operating pressures (0.2, 0.4, 0.6, 0.8, 1.0, and 1.2 MPa) and solute concentrations (10, 50, 100, 150, and 200 µg/ml^−1^) under three pH values (3.0, 5.5, and 8.0). The concentration of procyanidins in grape juice was adjusted by adding procyanidin extracts or purified water. The pH was adjusted by 25.0 mmol/L sodium hydroxide aqueous solution or 13.5 mmol/L hydrochloric acid aqueous solution to change the state of procyanidins.

### 2.6 Antioxidant activity determinations

Antioxidant activity is one of the important indexes to evaluate the quality of procyanidins. The ABTS method was selected to detect the antioxidant activity of samples (Arend et al., 2017). The ABTS radical‐scavenging activity of the samples was measured by the method described by Sachindra (Sachindra et al., 2007). ABTS radical solution was prepared by mixing 5 ml of ready‐to‐use ABTS solution with 100 ml of acetate buffer (0.05 M, pH 4.5) and five units of peroxidase and incubation at 37°C for 15 hr. The decolorisation of the ABTS radical solution was initiated by mixing 250 μl of ABTS solution with 25 μl of sample and incubation at 37°C for 1 hr in a 96‐well plate. To the sample blank, 250 μl of acetate buffer (pH 4.5, 0.05 M) was added instead of ABTS. ABTS solution without sample served as the control. The absorbance was measured at 405 nm twice, respectively, at the beginning and the end of the incubation period. Scavenging activity is calculated as:

(7) $$\text{Scavenging}\%=\left(1-\frac{A_{\text{sample}}-A_{\text{sample blank}}}{A_{\text{control}}}\right)\times 100$$

ABTS radical‐scavenging activity is represented as mg TBHQ equivalent/mg sample and can be calculated with TBHQ standard curve. The analyses were performed in triplicates.

### 2.7 Nanofiltration separation prediction model verification

The procyanidin concentration in a new grape juice sample was detected by high‐performance liquid chromatography (Agilent 1100, USA). A series of procyanidin concentrations were treated in the NF system under the operating pressures of 0.2, 0.4, 0.6, 0.8, 1.0, and 1.2 MPa to establish the NF separation prediction model. The *k* was calculated by the equations of Table 2 with the series of procyanidin concentrations, and then, the predicted rejections were fitted by Equation (6) with the value of *k*.

The experimental *R*_o_ was calculated according to Equation (1) and compared with the predicted value to analyze the applicability of NF separation prediction model.

### 2.8 Membrane morphology analysis

The NF membrane was washed with 25 mmol/L sodium hydroxide aqueous solution to remove contaminants. The polluted and cleaned membranes were detected by scanning electron microscope (*SEM*). From ZEISS MERLIN Compact ultra‐high‐resolution field emission scanning electron microscopy, test parameters were Mag 20.00 kx, WD 7.2 nm, EHT 10.00 kv, and scale 1 µm. Prior to *SEM* analysis, the membrane samples were air‐dried and subsequently coated with an ultrathin layer of carbon. Extreme care was taken when preparing the fouled and scaled membrane samples to ensure that the fouling and scaling layer remained intact.

## 3 RESULTS AND DISCUSSION

### 3.1 Nanofiltration Permeate flux

**Figure 1:**
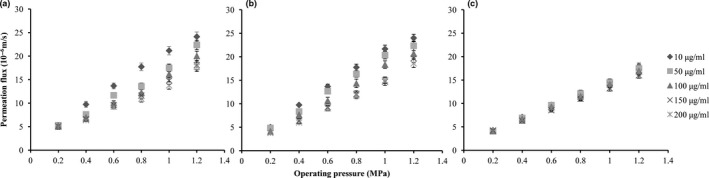
Influence of initial procyanidin feed concentration and operating pressure on the permeation flux, (a) pH 3.0, (b) pH 5.5, and (c) pH 8.0

The membrane permeate flux directly relates to the production efficiency, and the relationship between permeate flux and operating pressure provides the basis for solving the contradiction between the high production efficiency and serious membrane fouling. The results of NF membrane permeate flux under different pressures are shown in Figure 1. Under the condition of 25°C and pH 3.0, the permeate flux was increased linearly with the increase in the operating pressure and procyanidin rejection increased slightly with the increase in the operating pressure. However, the permeate flux was decreased with the increase in the concentration and this phenomenon became more obvious under higher operating pressures. As shown in Figure 1, the slopes of permeate flux‐operating pressure curves decrease with the increase in the solute concentration due to the changes in the solution and membrane properties, such as viscosity, conductivity, concentration polarization, and membrane pollution. This result was similar to the report by Cai (Cai, Hou, Lv, & Sun, 2017).

The effects of different pH on membrane permeate flux were analyzed (Figure 1). Procyanidins in grape juice existed in two states: ionic status and dissociative state. With the increase in pH, the proportion of the dissociative state increased accordingly, but the membrane permeate flux decreased because it was difficult for the solutes in the ionic status to pass through the NF membrane due to the charge effect (Ryzhkov & Minakov, 2016).

### 3.2 Effect of operating pressure on rejection

When the operating pressure of NF increased from 0.2 to 1.2 MPa, the rejections increased insignificantly. Meanwhile, the membrane flux was increased linearly on the whole and the NF concentration efficiency (the amount of water removed per unit time) was increased (Nakari, Pihlajamäki, & Mänttäri, 2016).

### 3.3 Effect of concentration on rejection

**Figure 2:**
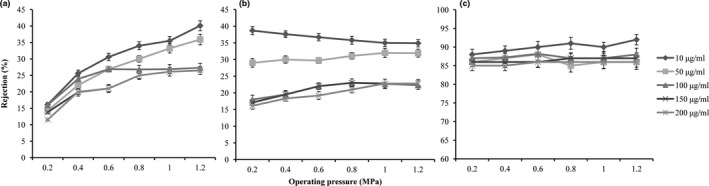
Effect of operating pressure on the rejection, (a) pH 3.0, (b) pH 5.5, and (c) pH 8.0

The effects of different concentrations from 10 to 200 µg/ml on the rejections during NF process were investigated. The rejection of procyanidins slowly decreased with the increase in the concentration (Figure 2). This result was consistent with the solution–diffusion theory (Paul, 2004; Wijmans & Baker, 1995). In NF process, procyanidin molecules accumulated in the boundary layer, so the local concentration of procyanidins in the boundary layer was much higher than that in the bulk. The increase in the solute concentration increased the permeable pressure difference and the solute could pass through the membrane pores, thus resulting in the decrease in the rejection. The solution–diffusion effect was increased under higher concentrations, which enhanced the membrane pollution and greatly affected the further separation. The solute rejection increased with the increase in the solution pH due to the Donnan effect between procyanidins and membrane surface charge.

### 3.4 Fitting mass transfer model

**Figure 3:**
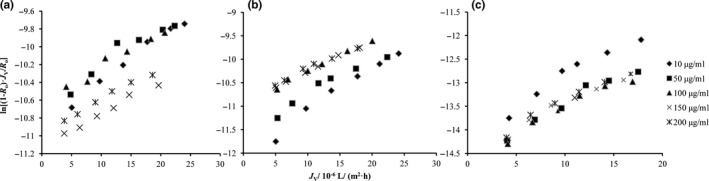
The correlation of ln[(1‐*R_o_*)·*J*_v_/*R_o_*] and *J*_V_ at different initial procyanidin concentrations, (a) pH 3.0, (b) pH 5.5, and (c) pH 8.0

The correlation between *J*_V_ and ln[(1‐*R_o_*)·*J*_v_/*R_o_*] was fitted by Equation (6) under a series of concentrations and operating pressures. The results under different pH values are shown in Figure 3. Then, *k* and ln[*DK*/*δ*] were calculated by Equation (6). The values of *k* and ln[*DK*/*δ*] in mass transfer model are shown in Table 1 (pH 3.0). The *k* of procyanidins increased with the concentration. The tendency was consistent with the solution–diffusion theory.

**Table 1:** The values of *k* and ln[*DK*/*δ*] at different initial procyanidin concentrations (pH 3.0)

*C*_o_(10^−7^ mol/L)	*k*(10^−6^ m/s)			ln[*DK*/*δ*]		
	pH 3.0	pH 5.5	pH 8.0	pH 3.0	pH 5.5	pH 8.0
0.21	19.80	10.58	8.28	−10.90	−12.08	−14.10
1.07	23.26	13.50	9.01	−10.66	−11.51	−14.59
2.13	26.11	14.73	9.65	−10.61	−10.94	−14.59
3.20	27.80	15.06	9.90	−11.12	−10.93	−14.48
4.27	27.70	16.37	9.81	−10.96	−10.82	−14.43

As the dissociative state of procyanidins was transformed into the dissociative and ionic coexistence, the rejections were changed dynamically. Dissociative procyanidins have the priority to enter NF membrane interface and are then dissolved to pass through NF membrane pores under the intermembrane pressure difference. Ionic procyanidins with NF membrane showed the charge effect (Table 1), and it was difficult for ionic procyanidins to pass through NF membrane, thus decreasing the mass transfer coefficient of procyanidins (pH 5.5). With the increase in the procyanidin concentration, the mass transfer coefficient was increased accordingly due to the solution–diffusion effect and the charge repulsion effect (Weng et al., 2016).

The NF membrane surface carries negative charge (Synder Filtration, USA). It is difficult for procyanidin anions to pass through the NF membrane due to the electrostatic repulsion between anions and the NF membrane. Therefore, the rejection increased accordingly. Based on the data in Table 1, at pH 8.0, the mass transfer coefficient of procyanidins in the ionic state was significantly lower than that of procyanidins in the dissociative state. In addition, ln[*DK*/*δ*] value was independent of the initial concentration of procyanidins, but it was related to the existence state of procyanidins.

### 3.5 Model verification

**Figure 4:**
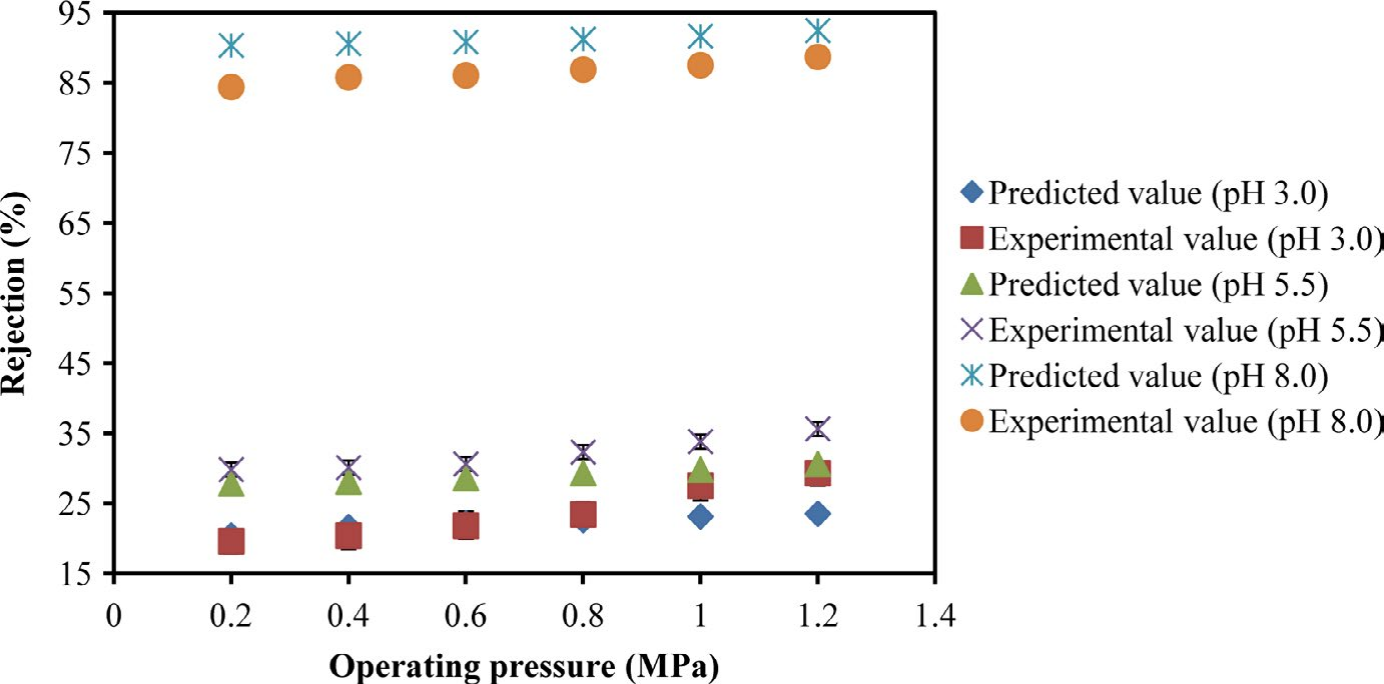
Comparison of the rejections between the experimental data and the predicted values of procyanidins with different pH values

The correlation between the procyanidin concentration and *k* was fitted by the exponential equation under different pH conditions (Table 2), and then, the experimental *R*_o_ was calculated with *J*_V_ and *C*_o_ according to Equation (6). The procyanidin concentration in fresh grape juice was 85.1 mg/ml, and the experimental values of rejection were obtained with different operating pressures and permeate fluxes. At pH 3.0 and 5.5, the experimental rejection was slightly higher than the predicted value, especially when the operating pressure was higher than 0.8 MPa (Figure 4) because some phenolic acids were competitively dissolved in membrane surface and led to the concentration polarization phenomenon. Therefore, it was difficult for procyanidin molecules to penetrate the membrane.

**Table 2:** The correlation of *k* and *C*_o_ with different pH

pH	Equations	*R* ^2^	ln[*DK*/*δ*]
3.0	$k=23.66C_{\text{o}}^{0.12}$	0.982	−10.85
5.5	$k=13.19C_{\text{o}}^{0.14}$	0.989	−11.26
8.0	$k=9.10C_{\text{o}}^{0.06}$	0.969	−14.44

At pH 8.0, the experimental rejection was slightly lower than the predicted value, unlike the results at the pH 3.0 and 5.5. Ionic state was the main existence state of procyanidins in solution, but its ionization level might be lower than other phenolic acids. Therefore, it is easy for the procyanidin molecule to penetrate the membrane.

### 3.6 Antioxidant activity determination

ABTS assays indicated that the antioxidant activity in the concentrate was significantly increased (*p* < 0.01). The concentration factor of procyanidins was 3.8. NF could efficiently separate the main bioactive compounds including phenolic compounds from grape juice. Phenolic compounds determine the quality of grape juice.

### 3.7 Membrane fouling of grape juice

**Figure 5:**
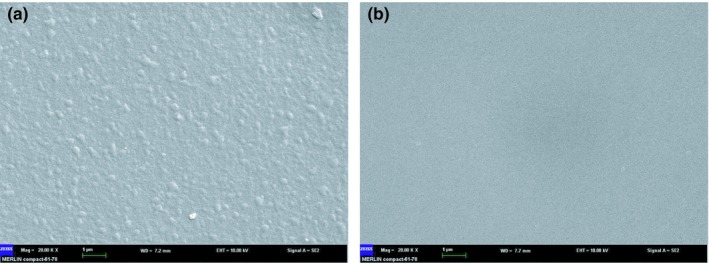
*SEM* image of NF membrane surface, (a) polluted membrane and (b) cleaned membrane

The morphology of the scaling layer confirmed the deposition of grape juice on the membrane surface (Figure 5a). The cake layer of grape juice on the membrane surface was easily cleaned (Figure 5b), and the membrane flux increased rapidly with washing time, suggesting that membrane fouling has redissolved. The good separation of grape juice was achieved by polyamide NF membranes, while the concentration efficiency was maintained.

## 4 CONCLUSION

Heat‐sensitive ingredients were concentrated by NF technology at normal temperature (15–27°C). The interaction force between procyanidins and NF membrane mainly involved the solution–diffusion effect and charge repulsion. Therefore, the rejection of procyanidins could be adjusted by changing the existence states of procyanidins. Mass transfer model was established on the basis of the solution–diffusion theory and Donnan effect to demonstrate the relationships between rejection and molecular existence parameters. *C*_o_ and pH were the main factors of the rejection. The *k* of procyanidins was directly related to the concentration under a fixed pH.

In recent years, NF studies were focused on the separation of glucose and ionic components based on the solution–diffusion theory and Donnan effect (Pérez et al., 2016; Wang et al., 2012). The exploration of the mass transfer mechanism for the NF separation of procyanidins provides the basis for improving NF separation of procyanidins. In the mass transfer model, electrical properties of procyanidins are the important parameter of *k*. The NF membrane surface carries negative charges. It is difficult for procyanidin anions to pass through the NF membrane due to the electrostatic repulsion between anions and the NF membrane. Therefore, the mass transfer coefficient increased accordingly. Then, the targeted rejection can be achieved by changing the existence state, concentration, and operating pressure.

Nanofiltration separation is an effective technique for the concentration of procyanidins from grape juice. In addition, the NF technology increases the utilization of agricultural products greatly and decreases the energy consumption.

## CONFLICT OF INTEREST

The authors have declared no conflict of interest.

## ETHICAL APPROVAL

The study did not involve any human or animal experimentation.

## References

[fsn31045-bib-0001] Arend, G. D. , Adorno, W. T. , Rezzadori, K. , Luccio, M. D. , Chaves, V. C. , Reginatto, F. H. , … José, C. C. (2017). Concentration of phenolic compounds from strawberry (*Fragaria X ananassa Duch*) juice by nanofiltration membrane. Journal of Food Engineering, 201, 36–41. 10.1016/j.jfoodeng.2017.01.014

[fsn31045-bib-0002] Banerjee, P. , & De, S. (2011). Modeling of nanofiltration of dye using a coupled concentration polarization and pore flow model. Separation Science and Technology, 46(4), 561–570. 10.1080/01496395.2010.534525

[fsn31045-bib-0003] Cai, M. , Hou, W. , Lv, Y. , & Sun, P. (2017). Behavior and rejection mechanisms of fruit juice phenolic compounds in model solution during nanofiltration. Journal of Food Engineering, 195, 97–104. 10.1016/j.jfoodeng.2016.09.024

[fsn31045-bib-0004] Dumpler, J. , & Kulozik, U. (2016). Heat‐induced coagulation of concentrated skim milk heated by direct steam injection. International Dairy Journal, 59, 62–71. 10.1016/j.idairyj.2016.03.009

[fsn31045-bib-0005] Geraldes, V. , Semião, V. , & Pinho, M. N. D. (2001). Flow and mass transfer modelling of nanofiltration. Journal of Membrane Science, 191(1), 109–128. 10.1016/S0376-7388(01)00458-6

[fsn31045-bib-0006] Guu, Y. K. , & Zall, R. R. (1992). Nanofiltration concentration effect on the efficacy of lactose crystallization. Journal of Food Science, 57(3), 735–739. 10.1111/j.1365-2621.1992.tb08084.x

[fsn31045-bib-0007] Hidalgo, A. M. , León, G. , Gómez, M. , Murcia, M. D. , Barbosa, D. S. , & Blanco, P. (2013). Application of the solution‐diffusion model for the removal of atrazine using a nanofiltration membrane. Desalination and Water Treatment, 51(10–12), 2244–2252. 10.1080/19443994.2012.734720

[fsn31045-bib-0008] Imbierowicz, M. , Troszkiewicz, M. , & Piotrowska, K. (2015). Heat effects of wet oxidation of glucose: A biomass model compound. Chemical Engineering Journal, 260, 864–874. 10.1016/j.cej.2014.08.084

[fsn31045-bib-0009] Khanal, R. C. , Howard, L. R. , & Prior, R. L. (2010). Effect of heating on the stability of grape and blueberry pomace procyanidins and total anthocyanins. Food Research International, 43, 1464–1469. 10.1016/j.foodres.2010.04.018

[fsn31045-bib-0010] Kyraleou, M. , Pappas, C. , Voskidi, E. , Kotseridis, Y. , Basalekou, M. , Tarantilis, P. A. , & Kallithraka, S. (2015). Diffuse reflectance Fourier transform infrared spectroscopy for simultaneous quantification of total phenolics and condensed tannins contained in grape seeds. Industrial Crops and Products, 74, 784–791. 10.1016/j.indcrop.2015.06.016

[fsn31045-bib-0011] Lecce, G. D. , Arranz, S. , Jáuregui, O. , Tresserra‐Rimbau, A. , Quifer‐Rada, P. , & Lamuela‐Raventós, R. M. (2014). Phenolic profiling of the skin, pulp and seeds of albariño grapes using hybrid quadrupole time‐of‐flight and triple‐quadrupole mass spectrometry. Food Chemistry, 145, 874–882. 10.1016/j.foodchem.2013.08.115 24128559

[fsn31045-bib-0012] Li, Y. , Qi, B. , Luo, J. , Khan, R. , & Wan, Y. (2015). Separation and concentration of hydroxycinnamic acids in alkaline hydrolyzate from rice straw by nanofiltration. Separation and Purification Technology, 149, 315–321. 10.1016/j.seppur.2015.06.006

[fsn31045-bib-0013] Lim, J. , Scholes, C. A. , Dumée, L. F. , & Kentish, S. E. (2014). Nanofiltration for the concentration of heat stable salts prior to mea reclamation. International Journal of Greenhouse Gas Control, 30, 34–41. 10.1016/j.ijggc.2014.08.020

[fsn31045-bib-0014] Maher, A. , Sadeghi, M. , & Moheb, A. (2014). Heavy metal elimination from drinking water using nanofiltration membrane technology and process optimization using response surface methodology. Desalination, 352, 166–173. 10.1016/j.desal.2014.08.023

[fsn31045-bib-0015] Montealegre, R. R. , Peces, R. R. , Vozmediano, J. L. C. , Gascueña, J. M. , & Romero, E. G. (2006). Phenolic compounds in skins and seeds of ten grape *Vitis vinifera* varieties grown in a warm climate. Journal of Food Composition and Analysis, 19, 687–693. 10.1016/j.jfca.2005.05.003

[fsn31045-bib-0016] Murthy, Z. V. P. , & Gupta, S. K. (1997). Estimation of mass transfer coefficient using a combined nonlinear membrane transport and film theory model. Desalination, 109(1), 39–49. 10.1016/S0011-9164(97)00051-9

[fsn31045-bib-0017] Nakari, O. , Pihlajamäki, A. , & Mänttäri, M. (2016). Permeability of dilute ionic liquid solutions through a nanofiltration membrane‐effect of ionic liquid concentration, filtration pressure and temperature. Separation and Purification Technology, 163, 267–274. 10.1016/j.seppur.2016.02.052

[fsn31045-bib-0018] Paul, D. R. (2004). Reformulation of the solution‐diffusion theory of reverse osmosis. Journal of Membrane Science, 241(2), 371–386. 10.1016/j.memsci.2004.05.026

[fsn31045-bib-0019] Pérez, L. , Escudero, I. , Arcos‐Martínez, M. J. , & Benito, J. M. (2016). Application of the solution‐diffusion‐film model for the transfer of electrolytes and uncharged compounds in a nanofiltration membrane. Journal of Industrial and Engineering Chemistry, 47, 368–374. 10.1016/j.jiec.2016.12.007

[fsn31045-bib-0020] Qiu, X. , & Yang, Q. (2010). Quantitative structure‐activity relationship between compound molecular characteristics and nanofiltration separation efficiency. Advanced Materials Research, 168–170, 1185–1188. 10.4028/www.scientific.net/AMR.168-170.1185

[fsn31045-bib-0021] Revilla, E. , & Ryan, J. M. (2000). Analysis of several phenolic compounds with potential antioxidant properties in grape extracts and wines by high‐performance liquid chromatography‐photodiode array detection without sample preparation. Journal of Chromatography A, 881(1–2), 461–469. 10.1016/S0021-9673(00)00269-7 10905728

[fsn31045-bib-0022] Ryzhkov, I. I. , & Minakov, A. V. (2016). Theoretical study of electrolyte transport in nanofiltration membranes with constant surface potential/charge density. Journal of Membrane Science, 520, 515–528. 10.1016/j.memsci.2016.08.004

[fsn31045-bib-0023] Sachindra, N. M. , Sato, E. , Maeda, H. , Hosokawa, M. , Niwano, Y. , Kohno, M. , & Miyashita, K. (2007). Radical scavenging and singlet oxygen quenching activity of marine carotenoid fucoxanthin and its metabolites. Journal of Agricultural and Food Chemistry, 55, 8516–8522. 10.1021/jf071848a 17894451

[fsn31045-bib-0024] Wang, R. , Li, Y. , Wang, J. , You, G. , Cai, C. , & Chen, B. H. (2012). Modeling the permeate flux and rejection of nanofiltration membrane separation with high concentration uncharged aqueous solutions. Desalination, 299(4), 44–49. 10.1016/j.desal.2012.05.014

[fsn31045-bib-0025] Weng, X. D. , Bao, X. J. , Jiang, H. D. , Chen, L. , Ji, Y. L. , An, Q. F. , & Gao, C. J. (2016). pH‐responsive nanofiltration membranes containing carboxybetaine with tunable ion selectivity for charge‐based separations. Journal of Membrane Science, 520, 294–302. 10.1016/j.memsci.2016.08.002

[fsn31045-bib-0026] Wijmans, J. G. , & Baker, R. W. (1995). The solution‐diffusion model: A review. Journal of Membrane Science, 107(1–2), 1884–21. 10.1016/0376-7388(95)00102-I

